# Clinicopathologic characteristics of secondary squamous cell carcinoma of head and neck in survivors of allogeneic hematopoietic stem cell transplantation for hematologic malignancies

**DOI:** 10.1038/s41409-018-0299-x

**Published:** 2018-08-20

**Authors:** Chakra P. Chaulagain, Kellie A. Sprague, Monika Pilichowska, Janet Cowan, Andreas K. Klein, Esha Kaul, Kenneth B. Miller

**Affiliations:** 1 Taussig Cancer Institute of Cleveland Clinic, Maroone Cancer Center, Cleveland Clinic Florida, 2950 Cleveland Clinic Blvd, Weston, FL 33331 USA; 2 Department of Medicine, Division of Hematology and Oncology, Tufts Medical Center and Tufts University School of Medicine, Boston, MA USA; 3 Department of Pathology, Tufts Medical Center and Tufts University School of Medicine, Boston, MA USA

**Keywords:** Stem cells, Haematopoietic stem cells, Bone marrow transplantation

## Abstract

The risk of late complications including secondary malignancies is increased in long-term survivors of allogeneic hematopoietic stem cell transplants (HSCT). There is limited literature on the biological behavior and clinical features of squamous cell carcinoma (SCC) of head and neck post-HSCT. We present the clinical and pathologic characteristics on six patients who were diagnosed with SCC while in remission following an allogeneic HSCT. Median follow-up was 8 years. Five patients (83%) developed SCC of tongue and one developed esophageal SCC. Five patients had oral chronic graft-versus-host disease (cGvHD). The conventional risk factors of alcohol, tobacco, and human papillomavirus were absent. The most common presenting finding was the new-onset focal oral pain and ulcerated plaques clinically indistinguishable from a flare of their oral cGvHD lesions. We demonstrated that the SCC in three patients was of donor origin.

## Introduction

Advances in hematopoietic stem cell transplants (HSCT) are curing an increasing number of patients with hematologic malignancies leading to an increase in the number of long-term survivors. However, survivors are developing late complications following the HSCT, including an increase in delayed cardiovascular morbidity, late pulmonary complications, and an increased risk of secondary solid cancers[1–3]. The risk of secondary solid cancers is increased both with total body irradiation (TBI) and non-TBI-based conditioning regimens [3, 4]. The advent of reduced-intensity conditioning (RIC) has greatly expanded the scope of HSCT but the risk of secondary malignancies after RIC HSCT is similar to patients who receive a myeloablative conditioning regimen [4]. In a large reported cohort of HSCT recipients, the oral cavity was one of the most common SCC sites accounting for 15% of all solid cancers [5]. We report on six patients who developed SCC and review the literature of reports of SCCs. This study and other small series have identified a strong association between oral SCC and chronic graft-versus-host disease (cGvHD) [6–9]. SCC formation by donor cells has also been noted in selected studies [9]. There is a limited literature on the SCC of head and neck in the survivors of HSCT. A better understanding of this group of SCC may help with prevention, early diagnosis, and improved therapy. The purpose of our analysis is to examine the risk factors, tumor characteristics, treatment, outcomes, and to address the donor or recipient origin of SCC in HSCT survivors.

## Patients and methods

In this institutional review board approved study, we reviewed records of adult patients who had undergone HSCT at Tufts Medical Center and subsequently developed SCC of head and neck during remission. A summary of clinical and pathology data is presented in Table 1. Human papillomavirus (HPV) testing of the tumor samples was performed both by immunohistochemistry and fluorescent in situ hybridization (FISH, Ventana INFORM HPV III Family 16 probe-B, Tucson, AZ, USA). We used descriptive statistical analysis comparing and contrasting our findings to the historical series published as of March 2017 (Table 2).

**Table 1:** Characteristics of secondary squamous cell carcinoma of head and neck in the survivors of hematopoietic stem cell transplantation for hematologic malignancies

Patient	1	2	3	4	5	6
**Age at SCC/sex**	49/M	48/M	43/F	66/F	49/M	44/M
**Latency of SCC (years)**	**3**	8	8	12	7	13
**Hematologic malignancy**	Transformed FL to DLBCL	AML	AML	CML	CML	CML
**Treatment before HSCT**	R-CVP, R-CHOP, and BEAM followed ASCT	Daunorubicin + Ara-C	Daunorubicin + Ara-C	Hydroxyurea, interferone, and splenic irradiation	Imatininb	Hydroxyurea, interferone, and splenic irradiation
**Type of HSCT/sex of donor**	RIC MSD/F	Ablative MSD/M	Ablative MSD/F	Ablative MSD/M	Ablative MSD/M	Ablative MSD/F
**Oral mucositis**	Severe needing TPN	Moderate	Moderate	Severe needing TPN	Moderate	Severe needing TPN
**Acute GVHD**	Skin, oral	Skin, GI	Skin	Oral, liver	Skin, GI	None
**Chronic GVHD location/grade**	Oral/extensive	None	Oral/extensive	Skin, oral/extensive	Oral/extensive	Skin, oral, and esophageal/extensive
**Chronic GVHD treatment**	C, P, Ph, and R	None	P, M, and Ph	P, M, and R	P, M, Ph, and R	P, M, Ph, R, and I
**Site of SCC**	L (T1) and R (T2) anterior tongue, the left floor of the mouth	Base of tongue and left tonsil	Left tongue	Right tongue	Right tongue	Distal esophagus
**Chief complaint/exam finding**	Focal pain in the areas of chronic irritation/ill-defined tender plaque with shallow ulcerations	New-onset neck swelling/exophytic growth at the base of the tongue	Focal pain/ulcerated ill-defined tender flat lesion	Focal pain/superficial tender ulcerated lesion	Focal pain/ exophytic ulcerated 2-cm lesion, and diffuse similar lesions nearby mucosa	Dysphagia and distal esophageal exophytic mass by endoscopy
**Stage of SCC/focality**	T2N0/stage II/multifocal	T1N2a/stage IVA/multifocal	T2N0/stage II/multifocal	T4aN2a/stage IVA/unifocal	pT2N1/stage III/multifocal	T3N1/stage III/no resection
**Grade**	3	2	2	2	2	2
**Resection margin**	+ for SCC, SCC in situ, and SSD	+ for SCC, SCC in situ, and SSD	+ for SCC and SSD	+ for SCC and SSD	+ for SSD	Patient declined resection
**LVI/PNI**	−/−	+/−	−/+	−/+	+/+	−/−
**Preceding lesions**	SD and SP	NPB	SD	SD	NPB	SD
**Treatment**	Neoadjuvant TPF, B/L hemiglossectomy, and floor of mouth excision	B/L tonsillectomy, base of tongue excision, CRT, and neck dissection	Partial glossectomy, neck dissection, and CRT at recurrence	Hemiglossectomy, neck dissection, and CRT with concurrent cetuximab	Hemiglossectomy and neck dissection	CRT and declined surgery
**Outcome**	2 local recurrence in 2 years and 4 surveillance biopsies showing SSD	Alive at 9 years without recurrence	Died after 4 years from multiple local recurrences, and pulmonary metastasis	Died of progressive SCC in 4 months	Alive at 15 months without recurrence	Alive at 18 months without recurrence

*Pt* patients; *SCC* squamous cell carcinoma; *ASCT* autologous hematopoietic stem cell transplantation; *FL* follicular lymphoma; *DLBCL* diffuse large B-cell lymphoma; *AML* acute myeloid leukemia; *CML* chronic myeloid leukemia; *R-CVP* rituximab, cyclophosphamide, vincristine, prednisone; *R-CHOP* rituximab, cyclophosphamide, doxorubicin, prednisone; *BEAM* BCNU, etoposide, cytarabine, melphalan; *RIC* reduced-intensity conditioning; *MSD* matched sibling donor; *M* male; *F* female; *TPN* total parenteral nutrition; *GI* gastrointestinal; *GVHD* graft-versus-host disease; *Ext* extensive; *C* cyclosporine; *P* prednisone; *M* mycophenolate mofetil; *Ph* photopheresis; *I* imatinib; *R* right; *L* left; *LVI* lymphovascular invasion; *PNI* perineural invasion; *SSD* severe squamous dysplasia; *SP* squamous papilloma; *NPB* no prior biopsy; *CRT* chemoradiotherapy; *B/L* bilateral; *TPF* docetaxel, carboplatin, and 5-flurouracil; *CRT* chemoradiation

**Table 2:** Characteristics of secondary squamous cell carcinoma of head and neck in the survivors of hematopoietic stem cell transplantation for hematologic malignancies (comparing our series with series from literature review)

Study	*N*	Oral cGvHD %	Prior dysplasia %	Multifocal/ metachronous SCC%	Tongue primary %	Median time from HSCT (years)	Risk factors (% of patients with alcohol use/tobacco use and HPV positivity)	Treatment	Recurrence %
Mawardi et al. [6]	15	96	19	28	56	6	4/15/NR	Surgery alone 67%	44 at median of 17 months
Chung et al. [7]	7	100	NR	33	100	8	0/0/NR		33
Chen et al. [8]	6	100	NR	None	50	10	0/0/0	NR	None at median of 3 years
Our series	6	83	67	67	83	8	0/0/0	Surgery alone 83%	50
Janin et al. [9]	4	100	NR	NR	NR	5–22	0/0/NR	Surgery alone 100%	All died of recurrence by 1 year
Jaguar et al. [11]	2	100	NR	NRp	50	13 and 15 years	0/0/NR	Surgery and radiation	No recurrence at 2 years
Reddy et al. [12]	3	33	NR	NR	33	1.5 years, 8 years, and 15 years	0/0/NR	Surgery alone	33
Szeto et al. [13]	2	100	NR	NR	100	2 and 6 years	0/0/NR	Surgery alone	50

*cGvHD* chronic graft-versus-host disease; *NR* not reported

### FISH analysis

**Fig. 1:**
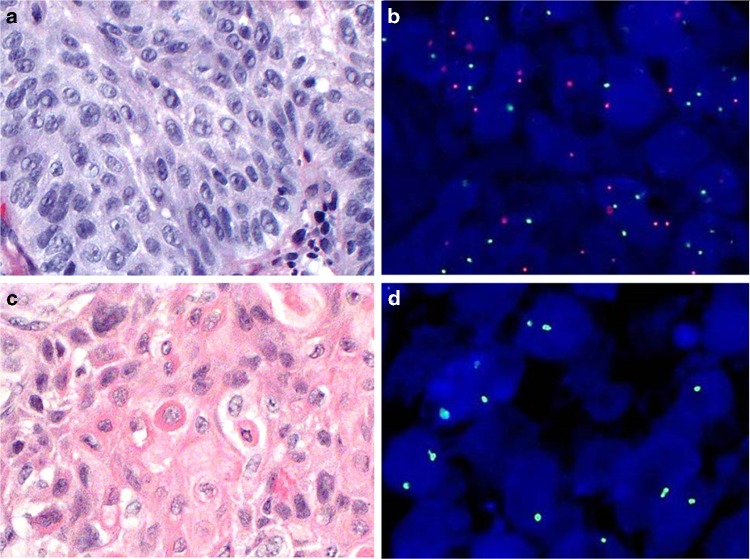
Histologic and cytogenetic FISH findings in squamous cell carcinoma in patients post HSCT. Patient A (H&E; ×400) showing moderately to poorly differentiated SCC and B showing the corresponding FISH (DAPI ×500). Note the red and green signals representing the presence of centromeric X and Y probes in the tumor cells (the patient was female, and the donor was male). Patient C (H&E; ×400) showing well-differentiated SCC and D showing the corresponding FISH (DAPI ×1000). Note the two green signals representing the presence of two centromeric X-chromosome probes in the tumor cells (the patient was male, and the donor was female)

FISH analysis was performed on SCC specimens of three sex-mismatched HSCT recipients. Four-micron sections from formalin-fixed, paraffin-embedded specimens were prepared using the aquarius tissue preteatment system (Cytocell). The probe used was to the centromeric regions of the X and Y chromosomes (Cytocell). In each area, the sex of the cells was noted, with female being two green signals and male being one red and one green signal (Fig. 1). The sex of the patient was confirmed from records after the completion of scoring.

## Results

From January 1993 to December 2016, six HSCT patients were diagnosed with SCC of the head and neck. Five patients received myeloablative conditioning (cyclophosphamide 60 mg/kg IV daily ×2 days and TBI 12,000 cGy in six divided fractions) and one patient received RIC with pentostatin and TBI as previously described [10]. Three patients received a sex-mismatched HSCT. All donors were HLA-matched (8/8) sibling donors, five patients received an unmanipulated bone marrow graft, and one received granulocyte colony-stimulating factor mobilized blood stem cells. Prophylaxis against GvHD consisted a short course of methotrexate and cyclosporine, as previously described [10]. All patients were followed by the bone marrow transplant physicians and the SCC was suspected and diagnosed based on new symptoms leading to a referral to ear–nose–throat (ENT) specialists for diagnostic biopsies. All patients were in a complete remission from their primary malignancy and were documented full donor hematopoietic chimera at the time of diagnostic biopsy. Secondary SCC was managed by the head and neck oncology multidisciplinary team.

The indication for the HSCT included chronic myeloid leukemia (*n* = 3), acute myeloid leukemia (*n* = 2), and transformed follicular lymphoma (*n* = 1). During the hospitalization for HSCT, three (50%) patients developed severe mucositis requiring total parenteral nutrition. The median age at the time of HSCT was 40 years (range: 31–54) and the median age at diagnosis of SCC was 48 years (range: 43–66). The median time from the HSCT to the diagnosis of SCC was 8 years (range: 3–13). Five patients (83%) developed oral SCC and one developed esophageal SCC. All but one patient (83%) had active oral cGvHD at the site of SCC and were on immunosuppressive therapy at the time of diagnosis of SCC for treatment of their cGvHD.

The tongue was the most common primary site for SCC (83%). The most common presenting symptom leading to the diagnosis of SCC was the new onset of increased focal oral pain (*n* = 4) and the most common exam findings were focal tenderness, ill-defined plaque with small ulcerations (*n* = 3), and exophytic growth (*n* = 3). The nonexophytic oral SCC lesions were clinically indistinguishable from the cGvHD flare lesions characterized by focal lichenification, inflammation, induration, erythema, ulceration, and plaque formation. Two patients (33%) were diagnosed with concurrent but separate primary SCC. Though the sizes of the primary tumor were relatively small (mostly T1 or T2), nodal metastasis was common (*n* = 4) with extracapsular extension in one patient. Other high-risk tumor-specific features included involvement of the resection margin (*n* = 4) and the presence of lymphovascular invasion (LVI) or perineural invasion (PNI, *n* = 4). The surgical pathology specimens in four out of five (80%) patients with oral SCC had multifocal invasive SCC, scattered foci of high-grade squamous dysplasia, and multiple foci of carcinoma in situ. Both the original SCC and the recurrent SCC lesions contained multifocal moderate-to-high-grade squamous dysplasia in the tissue sections uninvolved and away from SCC foci. Four patients (67%) had moderate-to-high-grade squamous dysplasia ranging 1–5 years prior to the subsequent biopsy confirmation of invasive SCC. Only one patient had a remote history of alcohol and tobacco use. All the SCC samples tested negative for HPV. Three patients developed recurrences and two died of locally aggressive and metastatic SCC. All three sex-mismatched SCC samples (patients 1, 4, and 6) assessed by FISH were found to be of donor origin (Fig. 1 shows the representative H&E and FISH findings). In each case, the phenotype of the cells in the control region (areas of tissue section uninvolved by the SCC) matched the patient’s identified sex.

## Discussion

Our study demonstrates that the SCC of the oral cavity in patients post HSCT presents with different clinical, pathological, and prognostic features compared to the SCC in the non-HSCT population. Based on our series and the series of others [6–9, 11–13] (Table 2), secondary oral SCC post HSCT presents with the following unique features: (1) high rate of cGvHD at the site of SCC, (2) presence of antecedent squamous dysplasia (19–67%), (3) tongue is the most common primary site, (4) multifocal or metachronous SCC is common (28–67%), (5) the median latency is approximately 8 years (range: 1.5–15 years) from HSCT, (6) low prevalence of alcohol, smoking, and HPV, (7) surgery was the only treatment modality utilized in the majority, and (8) high recurrence rate (range: 33–50%) often leading to early death.

Though exposure to tobacco and or alcohol, or active use, was present in prior studies, none of our patients had a history of either tobacco or alcohol use (Table 2).  This contrasts to the SCC of head and neck seen in non-HSCT patients, where smoking and tobacco use is attributed to the etiology of the  majority (75%) of SCC, suggesting different mechanisms of oncogenesis [14]. HPV is also strongly associated with (up to 60%) the SCC of the oropharynx in non-HSCT population, but none of our SCC samples tested positive for HPV, indicating that HPV does not play a vital role for oncogenesis of SCC in HSCT survivors [15]. Due to the known association of HPV in non-HSCT populations, some have proposed HPV vaccination in long-term survivors of HSCT using a quadrivalent HPV vaccine (contains serotypes 6, 11, 16, and 18) [16]. This strategy may not be effective for the prevention of SCC of head and neck in this patient’s population. Though the role of Epstein–Barr virus has been proposed, its association with SCC post SCT has not been established. Herpes simplex virus, varicella zoster virus, and cytomegalovirus are the frequent causes of mucosal inflammation and morbidity in HSCT survivors but their role in causation of SCC is also unknown.

The well-defined tumor-specific high-risk predictors in the SCC of the oral cavity include positive resection margins, PNI, LVI, and nodal metastasis (particularly with extracapsular spread) [17]. Our study examined these variables and underscored a high frequency of these adverse risk factors in the surgical specimens. Our study also revealed a high frequency of dysplasia in noncancerous oral mucosa suggesting a global oncogenic effect (field carcinogenesis). Furthermore, the diagnosis of SCC was often (67%) preceded by the diagnosis of squamous dysplasia by 1–5 years prior to the subsequent diagnosis of SCC. These findings support that the carcinogenesis in this setting is a prolonged multistep process likely related to accumulation of a series of mutations in response to the various toxic exposures (e.g., radiation, chemotherapy, and infections) and immunologic factors (cGvHD, immunosuppressive agents) specific to HSCT. Lichenoid lesions are the characteristic features of oral cGvHD. Moreover, patients with the idiopathic form of oral lichen planus have an increased risk or oral SCC [18]. The development of oral SCC of donor origin may reflect the homing of donor stem cells to the sites of inflammation. The finding of SCC in recipients of HSCT has been previously reported. It remains unclear if the transplanted donor stem cells give rise to the SCCs. It is possible that donor stem cells fuse with recipient epithelial cells, resulting in a mosaic genetic pattern of both donor and recipient cells [9].

In keeping with prior observations, our study also confirms that cGvHD requiring prolonged immunosuppressive therapy (Table 1) has a strong association with the development of SCC. The process by which cGvHD facilitates carcinogenesis remains unclear. The longer duration of cGvHD, and thereby longer duration of immunosuppressive therapy, and use of azathioprine as an immunosuppressive agent have previously been linked with the etiology of SCC of oral cavity [19]. However, none of our patients had received azathioprine. It is unclear that preventing severe cGvHD or reducing the duration of exposure to immunosuppressive agents will decrease the incidence of SCC in HSCT survivors.

The optimal management of SCC of head and neck in HSCT survivors is unknown. They are more likely to receive surgery as the sole therapy and less likely to receive standard definitive CRT even if it is indicated (Tables 1 and 2). Five patients (1–6) tolerated CRT poorly due to a flare of cGvHD resulting in interruption and noncompletion of the planned CRT. Patient 2 had no oral cG*v*HD and tolerated CRT and has no evidence of SCC. The absence of cG*v*HD may predict the completion of CRT and possibly better survival and the presence of cGvHD at the SCC site may predict noncompletion of the definitive therapy, progressive and/or recurrent disease, and early death. In a prior series, the main reason for withholding radiotherapy was the theoretical concern for further increasing the risk of carcinogenesis in the oral mucosa, which underscores the hesitancy on behalf of the clinicians to utilize potentially curative CRT in these patients [6]. Our data suggest that some patients can receive additional chemoradiotherapy for control of SCC.

The SCC in three sex-mismatched recipients originated from the donors. Our observation further supports the hypothesis that the donor-derived multipotent bone marrow hematopoietic or mesenchymal stem cells have the potential to home to the sites of mucosal injury from the cGVHD with the ability to eventually transform to SCC [9, 20, 21]. The complexity of this underlying mechanism of epithelial neoplasia from the donor cells in long-term survivors of HSCT is not yet defined; however, induction of cell fusion and/or incorporation of the pluripotent bone marrow elements into cancer cells by adopting the phenotype of the cancer microenvironment (development of mimicry) have been suggested [9, 20, 21]. It is unknown if the sex-mismatch, related versus unrelated donor status and other donor variables can also influence this process.

In conclusion, our report demonstrated that the SCC of oral cavity is a challenging secondary malignancy in the long-term survivors of HSCT due to its aggressive clinical behavior and poor outcome. Many of the SCC lesions are clinically indistinguishable from lesions of the cGvHD flare and the most common presentation is the new-onset focal oral pain from a nonhealing ulcerated plaque or an exophytic lesion. Any nonhealing oral lesion should be biopsied to rule out SCC. Since SCC in this setting is difficult to diagnose based on the expertise of transplant providers alone, survivors of HSCT should have a periodic comprehensive oral examination by the oral surgeon from the head and neck multidisciplinary oncology team and any suspicious lesions should be biopsied. Recently published recommendations for screening and preventive practices for long-term survivors of HSCT address the need for the long-term follow-up and cancer screening in survivors of HSCT [22, 23]. A multidisciplinary approach is needed to evaluate patients with oral squamous dysplasia to be followed closely for the development of SCC. Due to a high rate of recurrence, vigilance is also needed after therapy is completed. Patients, providers, and family members of the HSCT survivors should be aware of this serious late complication. HSCT patients with oral cGvHD should be evaluated by oral medicine physicians and have periodic ENT examinations.
